# Supplementary material for: Exploiting nonaqueous self-stratified electrolyte systems toward large-scale energy storage
Source: Nat Commun. 2023 Apr 20;14:2267. doi: 10.1038/s41467-023-37995-8 (PMC10119102; doi:10.1038/s41467-023-37995-8)
Supplement: Supplementary file 1 — Supplementary Infomation [file 41467_2023_37995_MOESM1_ESM.pdf]

Supplementary Information

# Exploiting Nonaqueous Self-Stratified Electrolyte Systems toward Large-Scale Energy Storage

**Wang et al.**

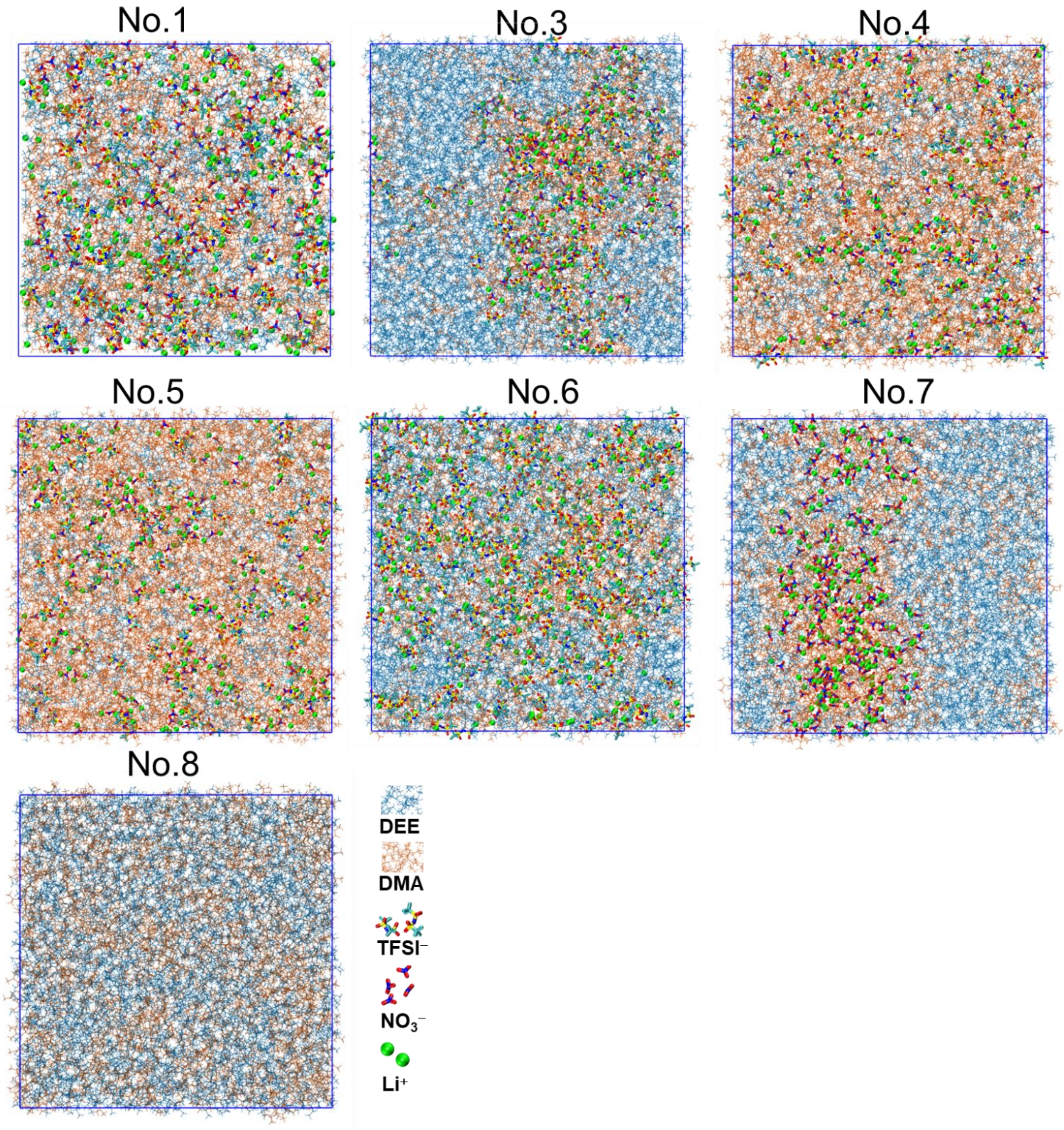


**Supplementary Figure 1.** The snapshots of the MD simulation of different systems, in which No.3 and No.7 appeared to have apparent phase separation while others demonstrated a homogeneous phase. This is consistent with the experimental phenomenon in Figure 1a.

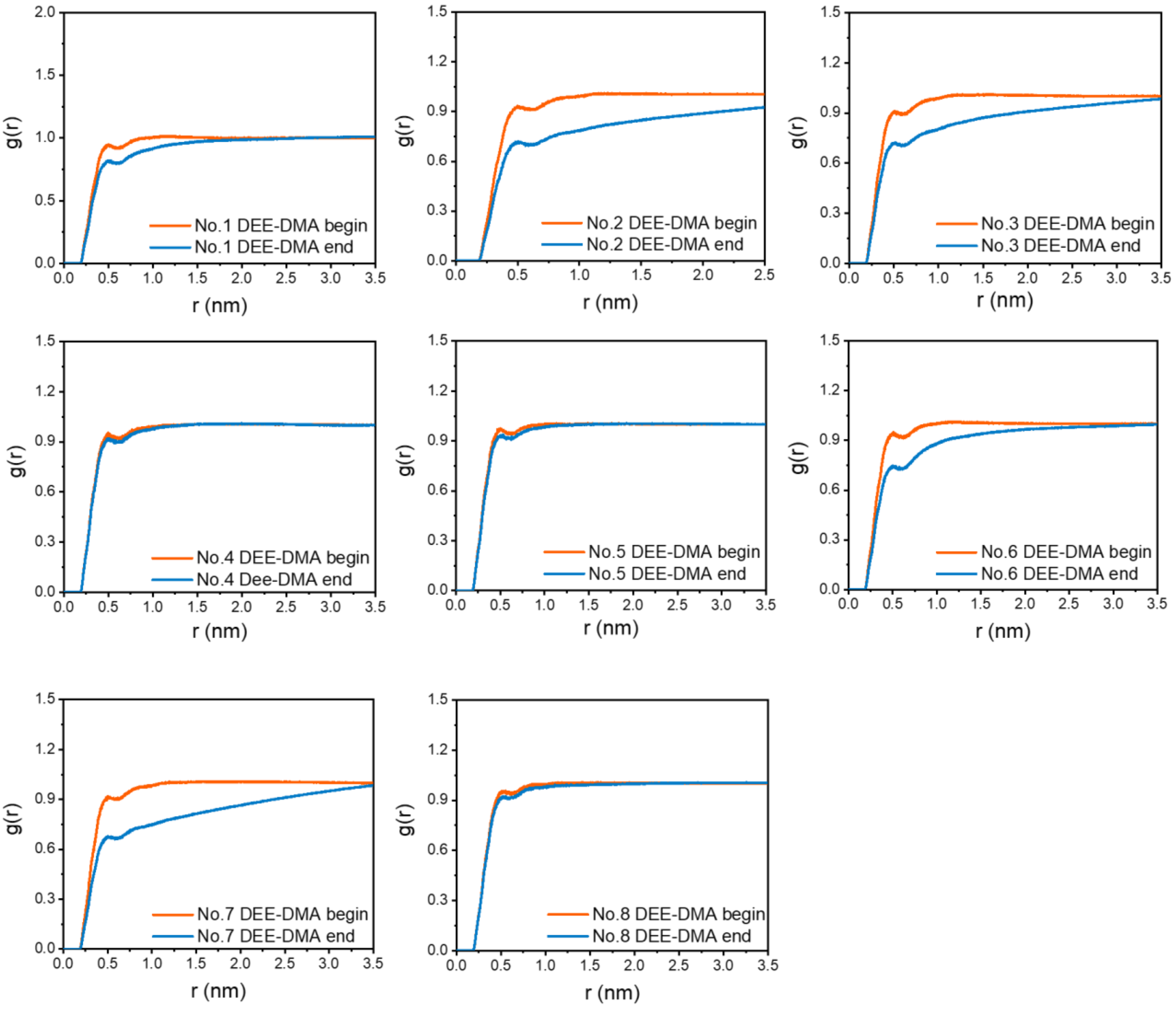


**Supplementary Figure 2.** The DEE-DMA radial distribution function at the beginning and end of MD simulations in different systems.

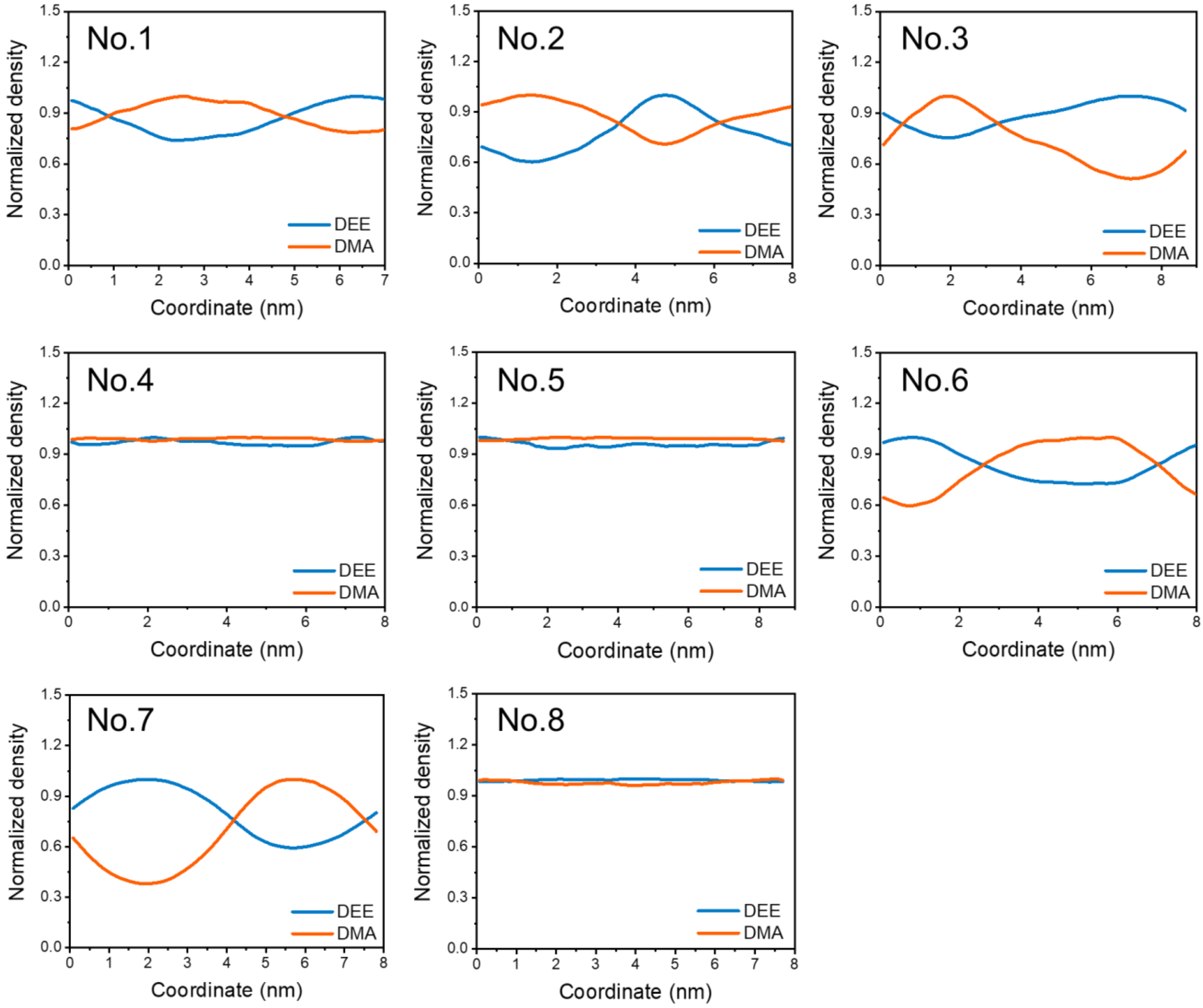


**Supplementary Figure 3.** The normalized spatial density distribution of DEE and DMA in different systems.

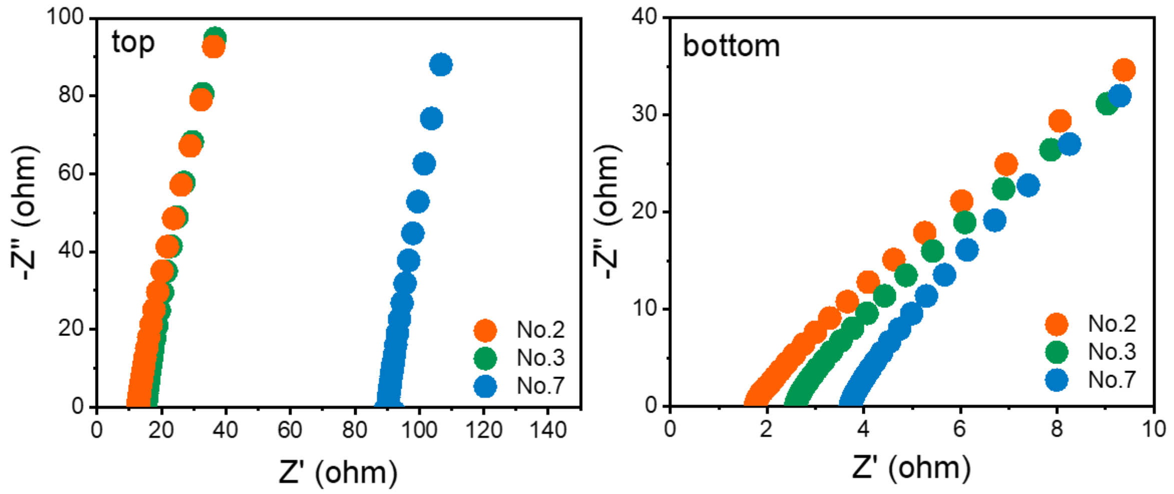


**Supplementary Figure 4.** Electrochemical impedance spectra of the No.2 electrolyte in the top phase and bottom phase, respectively.

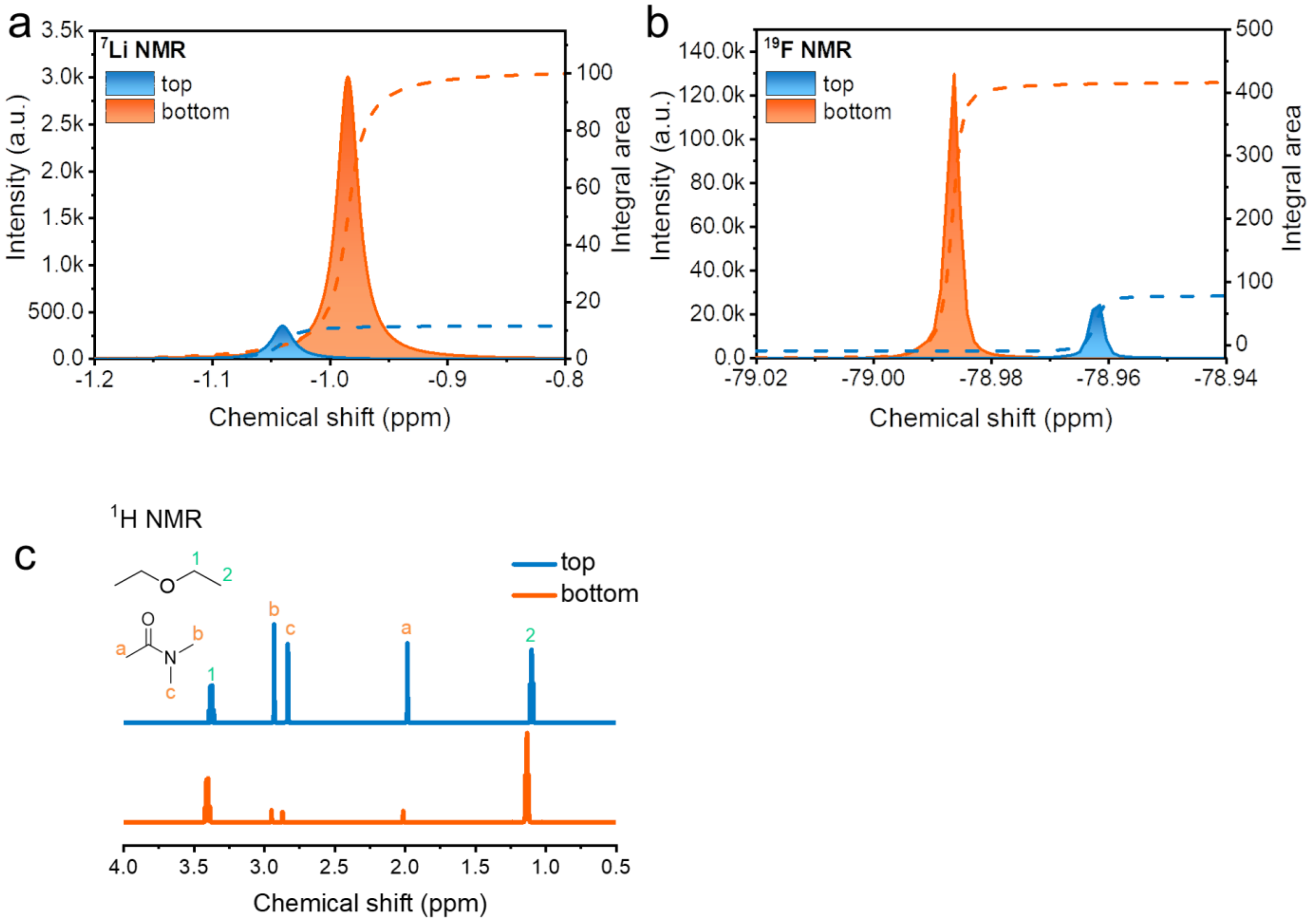


**Supplementary Figure 5.** $^{7}$Li NMR spectra (a) and $^{19}$F NMR (b) of the top and bottom phases, the dashed lines in them are the integral curves of the spectra. (c) $^{1}$H NMR spectra of the top and bottom phases.

The integral area of the NMR signal peak has a linear relationship with its concentration. Therefore, according to the results demonstrated in Figure S5d,

$$C_{DEE-T} = 7.4C_{DMA-T} \qquad (1)$$

$$C_{DEE-B} = 1.0C_{DMA-B} \qquad (2)$$

$C_{DEE-T}$ and $C_{DEE-B}$ are the concentration of DEE in the top and bottom phases, respectively; $C_{DMA-T}$, and $C_{DMA-B}$ are the concentration of DMA in the top and bottom phases, respectively.

In the No.2 system, the total volume of electrolyte is 3 ml, of which the top phase is 1.1 ml, and the bottom phase is 1.9 ml. The solvent mole in the electrolyte system is 30.02 mmol, of which DEE is 19.

23 mmol. These lead to the following relationship:

$$1.1C_{DEE-T} + 1.1C_{DMA-T} + 1.9C_{DEE-B} + 1.9C_{DMA-B} = 30.02 \quad (3)$$

$$1.1C_{DEE-T} + 1.9C_{DEE-B} = 19.23 \quad (4)$$

Combined equations (1) to (4), we can obtain that:

$$C_{DEE-T} = 8.67\ mM$$

$$C_{DMA-T} = 1.17\ mM$$

$$C_{DEE-B} = 5.10\ mM$$

$$C_{DMA-B} = 5.00\ mM$$

Therefore, the moles of solvent in the top and bottom phases can be obtained.

$$n_{DEE-T} = 9.54\ mmol$$

$$n_{DMA-T} = 1.29\ mmol$$

$$n_{DEE-B} = 9.69\ mmol$$

$$n_{DMA-B} = 9.50\ mmol$$

For the lithium salts concentration:

$$1.1C_{Li-T} + 1.9C_{Li-B} = 2 \quad (5)$$

$$99C_{Li-T} - 100C_{Li-B} = 0 \quad (6)$$

$$1.1C_{TFSI-T} + 1.9C_{TFSI-B} = 1 \quad (7)$$

$$470C_{TFSI-T} - 50C_{TFSI-B} = 0 \quad (8)$$

Therefore, the moles of lithium salts in the top and bottom phases can be obtained.

$$n_{Li-T} = 0.11\ mmol$$

$$n_{Li-B} = 1.89\ mmol$$

$$n_{TFSI-T} = 0.10\ mmol$$

$$n_{TFSI-B} = 0.89\ mmol$$

$$n_{NO3-T} = 0.01\ mmol$$

$$n_{NO3-B} = 0.99\ mmol$$

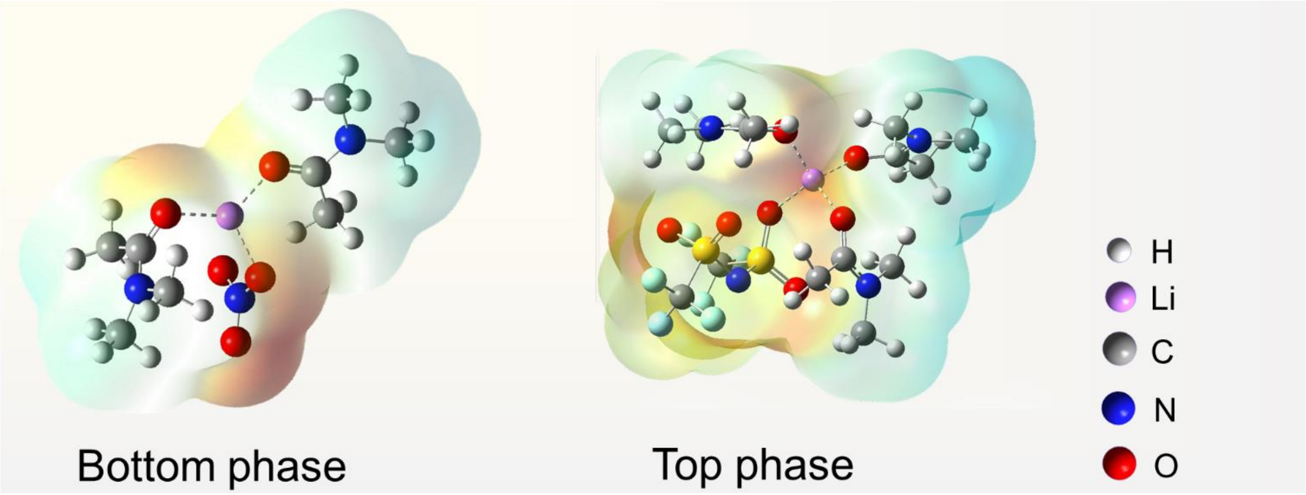


**Supplementary Figure 6.** Solvation structure of $Li^{+}$ in the top and bottom phases after DFT optimization and their electrostatic potential distribution maps.

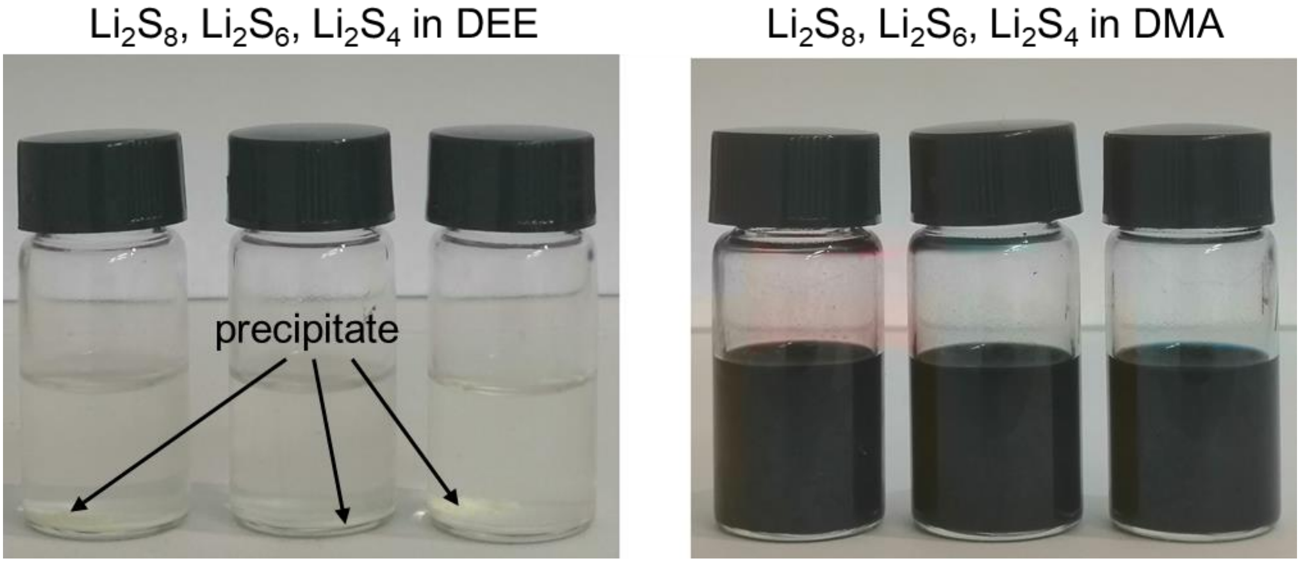


**Supplementary Figure 7.** Dissolution photograph of 10 mM lithium polysulfides ($Li_2S_8$, $Li_2S_6$, $Li_2S_4$) in DEE and DMA obtained by stirring stoichiometric amounts of $Li_2S$ and sulfur for 24 h.

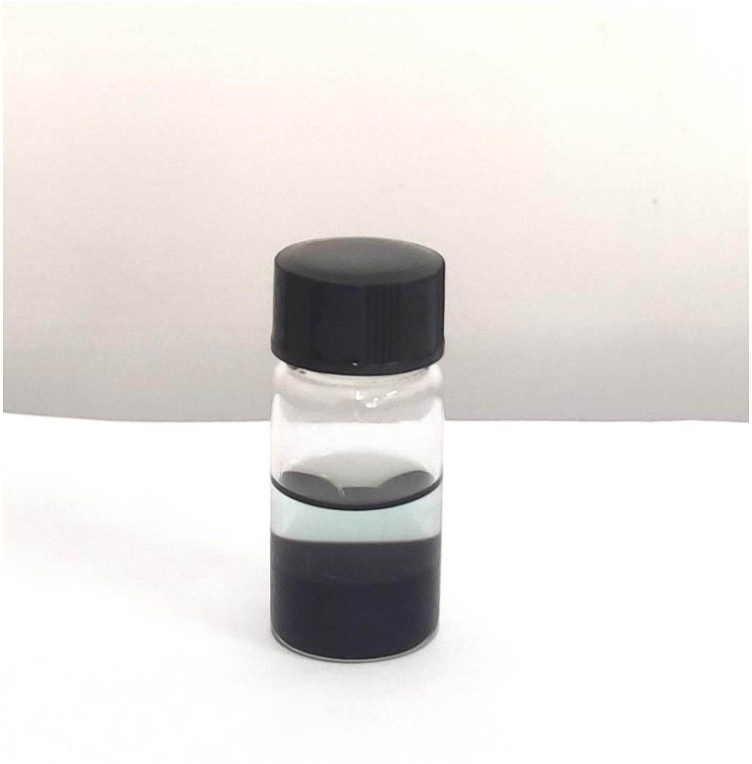

**Supplementary Figure 8.** The digital photograph of $Li_2S_8$ in DMA-DEE biphasic system, in which the top phase exhibits a slight blue color.

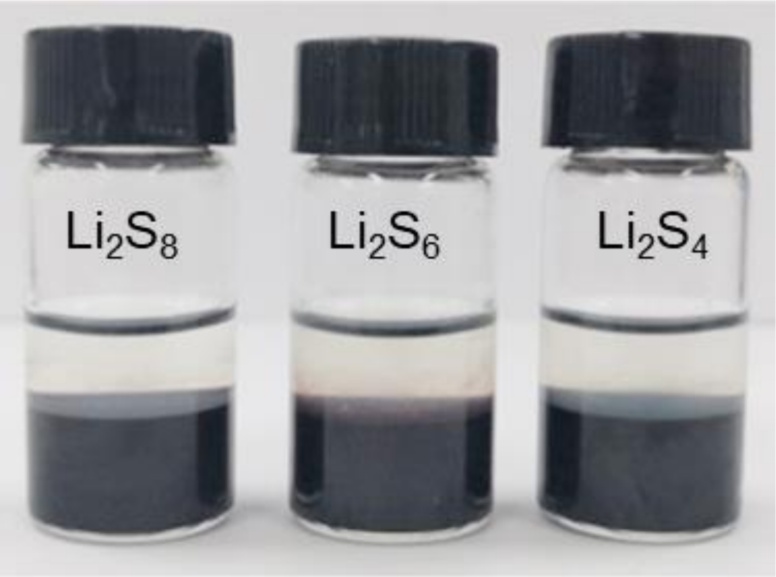


**Supplementary Figure 9.** The digital photograph of lithium polysulfides in DMA-DEE biphasic system after adding 0.5 mM TEMPO.

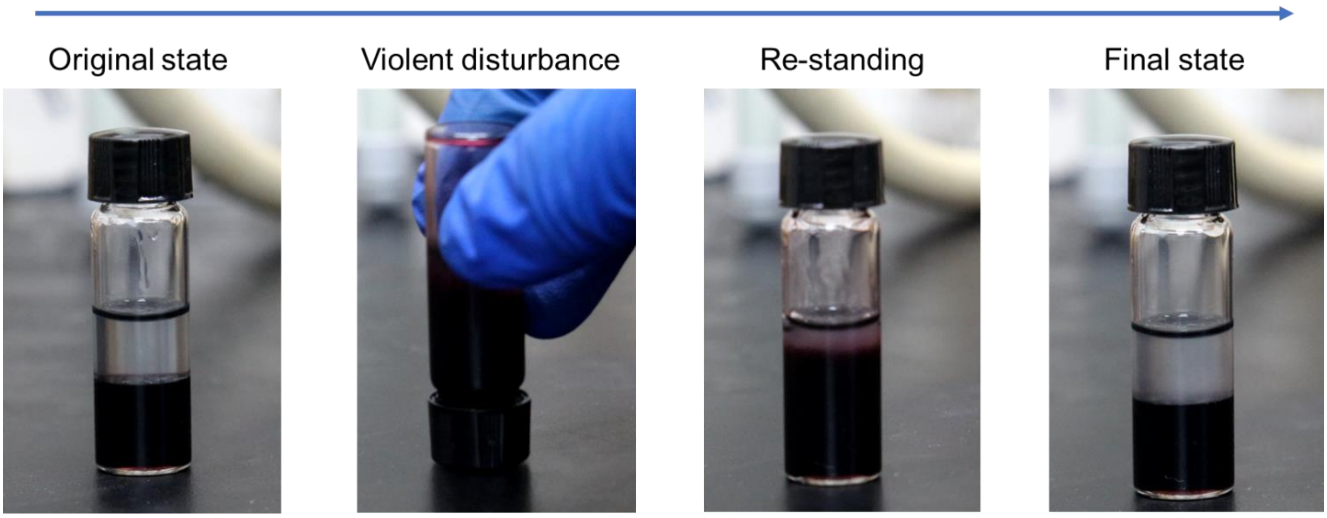


**Supplementary Figure 10.** The spontaneous recovery process of biphasic electrolyte system after external disturbance. 10 mM of $Li_2S_8$ was dissolved in the electrolyte.

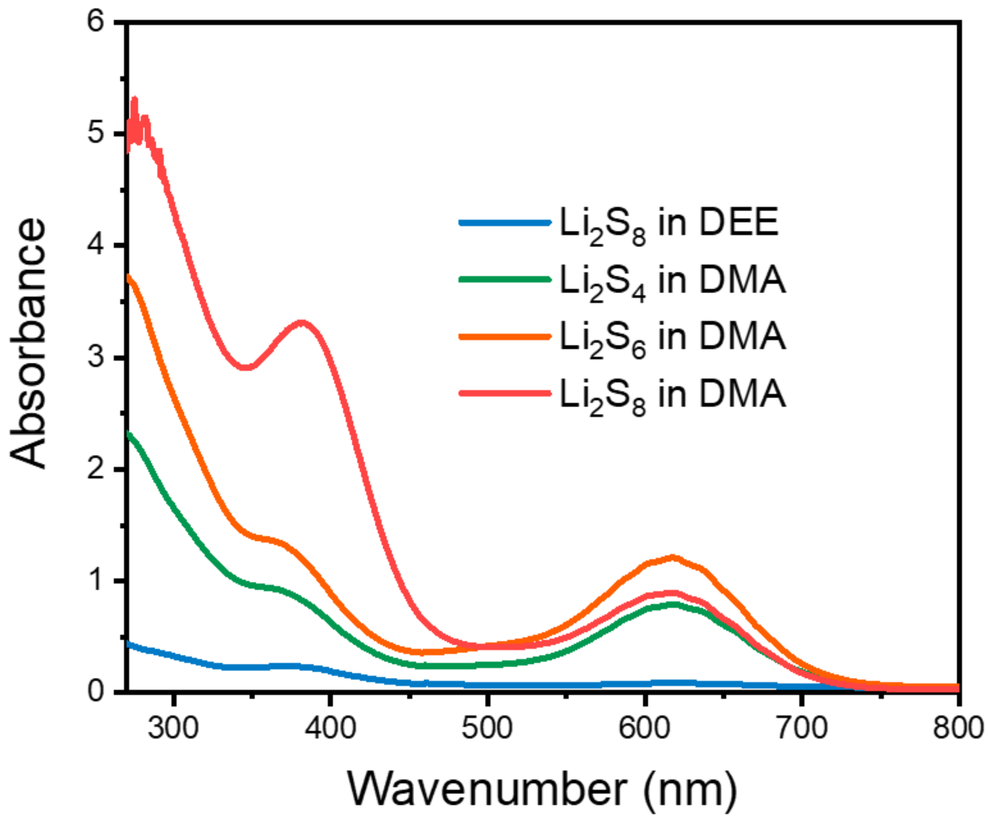


**Supplementary Figure 11.** The UV/Vis spectrum of 5 mM lithium polysulfides ($Li_2S_8$, $Li_2S_6$, $Li_2S_4$) in DEE and DMA.

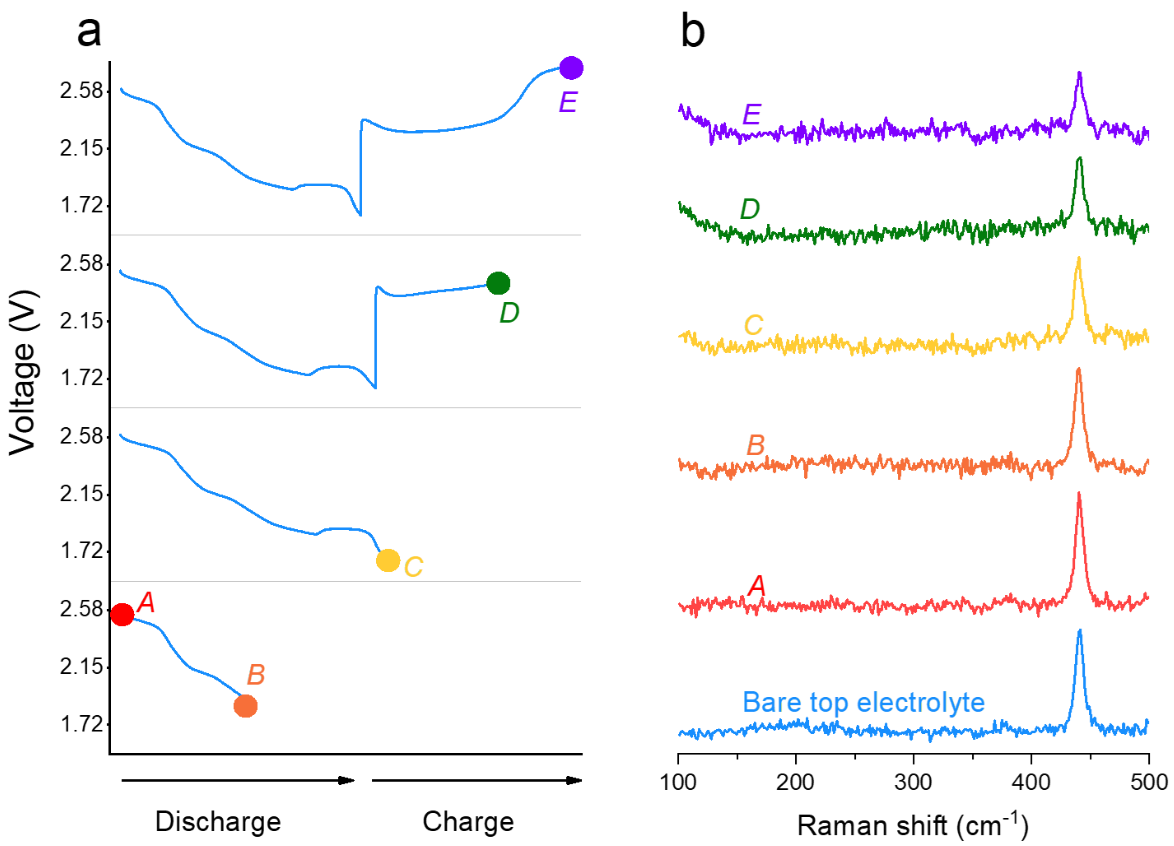


**Supplementary Figure 12.** The ex situ Raman tests of the top phase electrolyte in Li-S BSBs during discharge/charge. The Raman spectra of the electrolyte in the top phase of BSBs with different states of charge (a) are summarized in the right (b).

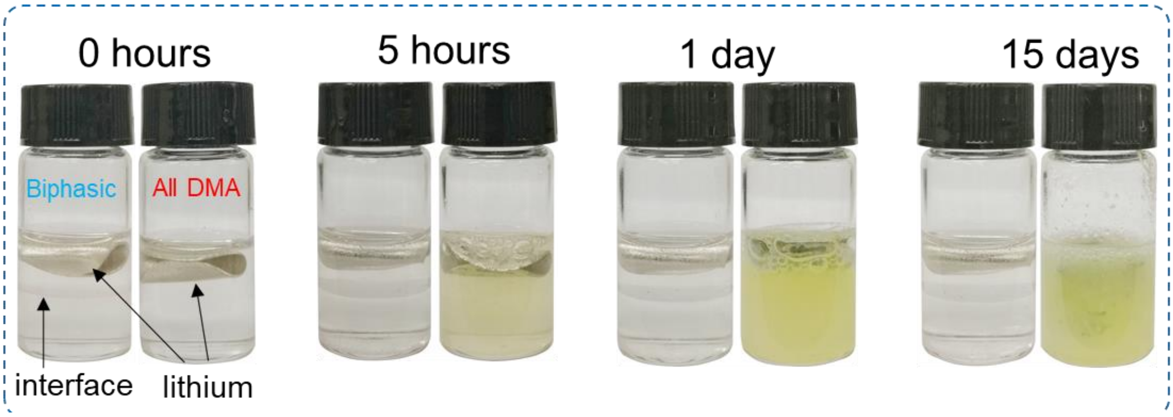


**Supplementary Figure 13.** The stability of metallic lithium in the top phase of the No.2 biphasic system and pure DMA.

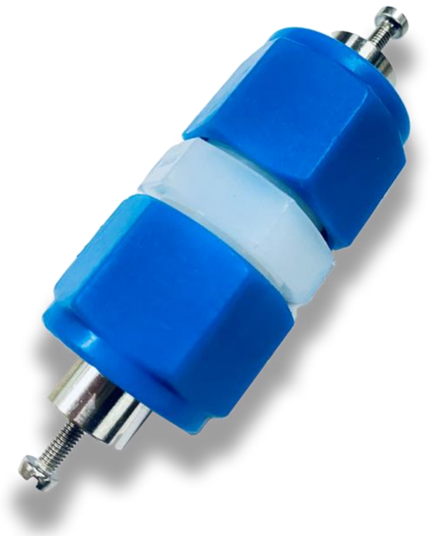

**Supplementary Figure 14.** The digital photograph of the well-designed Swagelok cell.

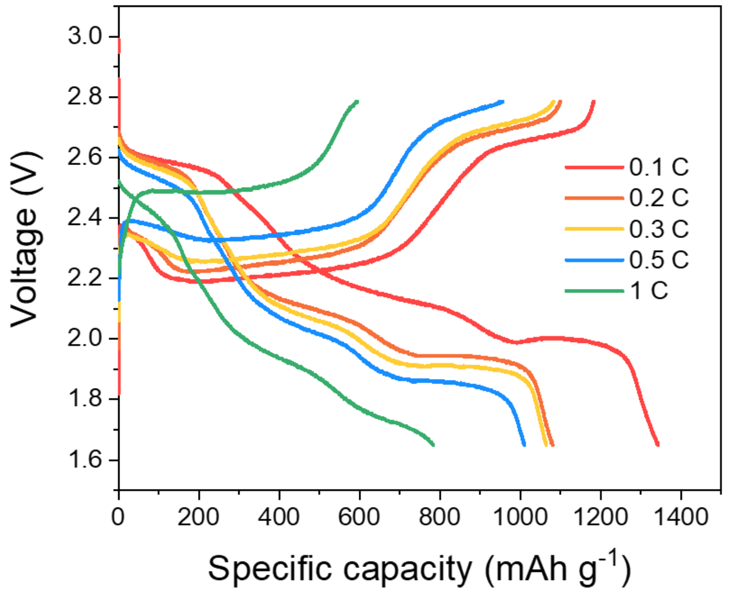


**Supplementary Figure 15.** The discharge/charge profiles of Li-S BSB at various current densities.

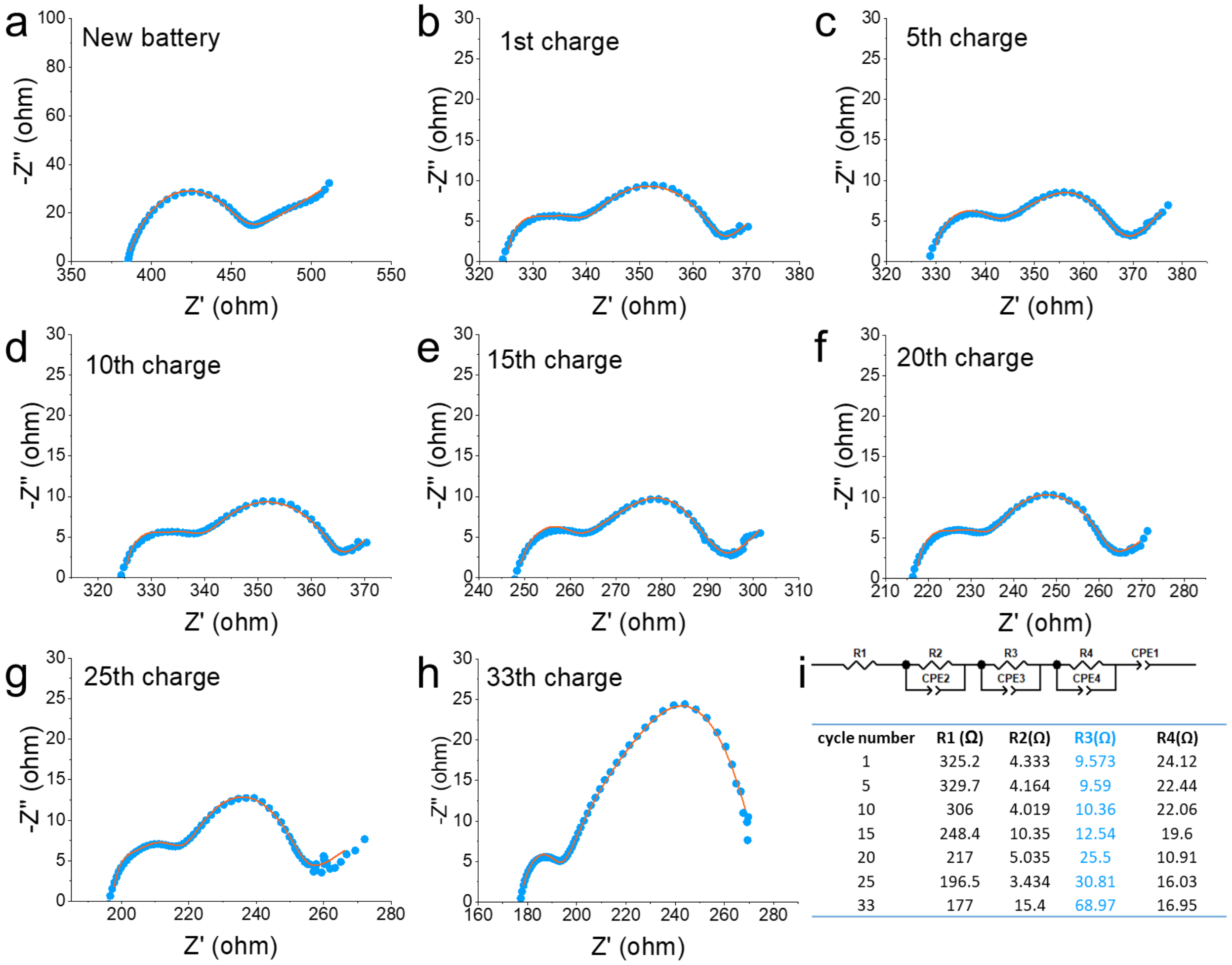


| cycle number | R1 (Ω) | R2(Ω) | R3(Ω) | R4(Ω) |
|---|---|---|---|---|
| 1 | 325.2 | 4.333 | 9.573 | 24.12 |
| 5 | 329.7 | 4.164 | 9.59 | 22.44 |
| 10 | 306 | 4.019 | 10.36 | 22.06 |
| 15 | 248.4 | 10.35 | 12.54 | 19.6 |
| 20 | 217 | 5.035 | 25.5 | 10.91 |
| 25 | 196.5 | 3.434 | 30.81 | 16.03 |
| 33 | 177 | 15.4 | 68.97 | 16.95 |

**Supplementary Figure 16.** (a)-(h) EIS of Li-S BSBs at different cycle numbers and their corresponding fitting results. (i) The equivalent electrical circuit for fitting EIS data after battery cycle and fitting results.

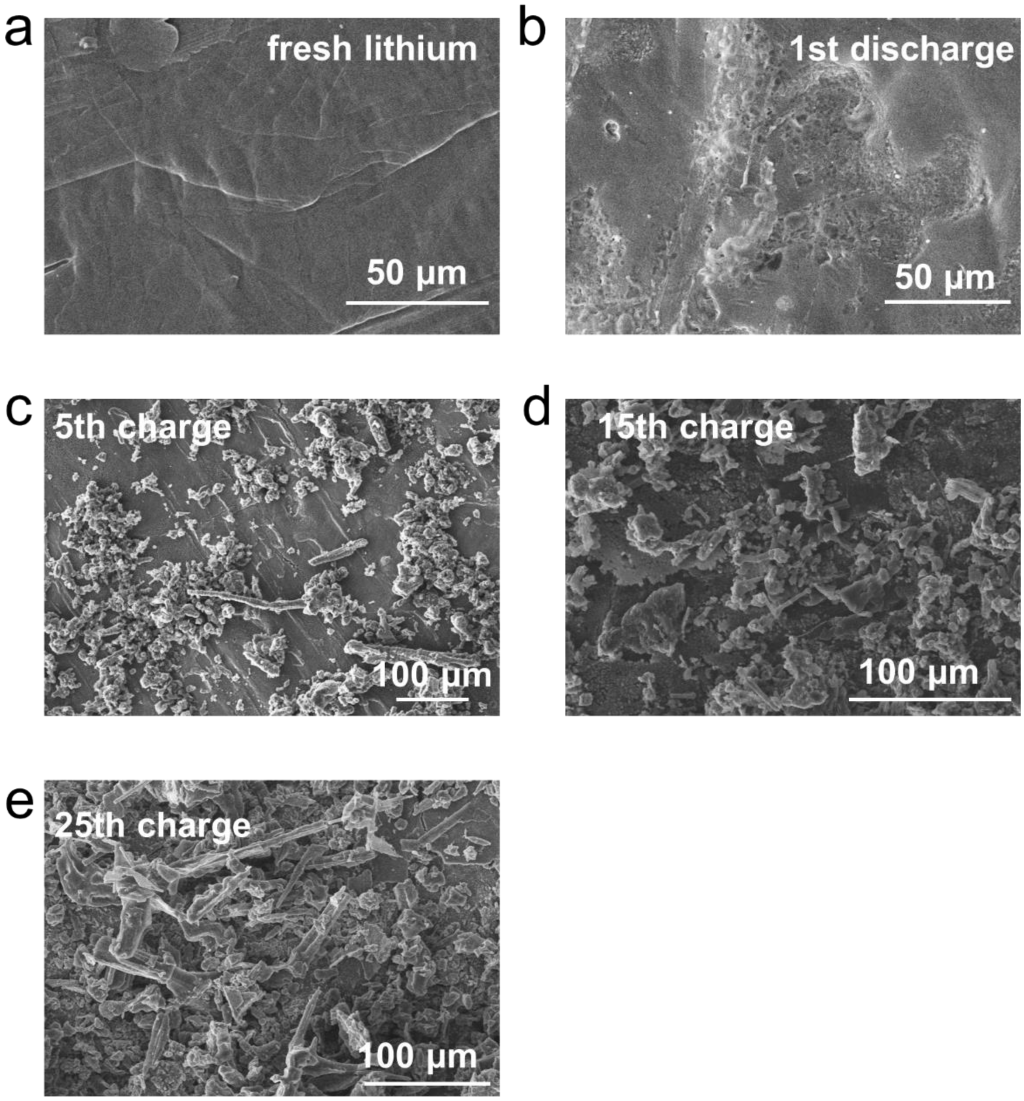


**Supplementary Figure 17.** (a)-(e) The surface morphology of lithium anode in Li-S BSBs at different cycle numbers.

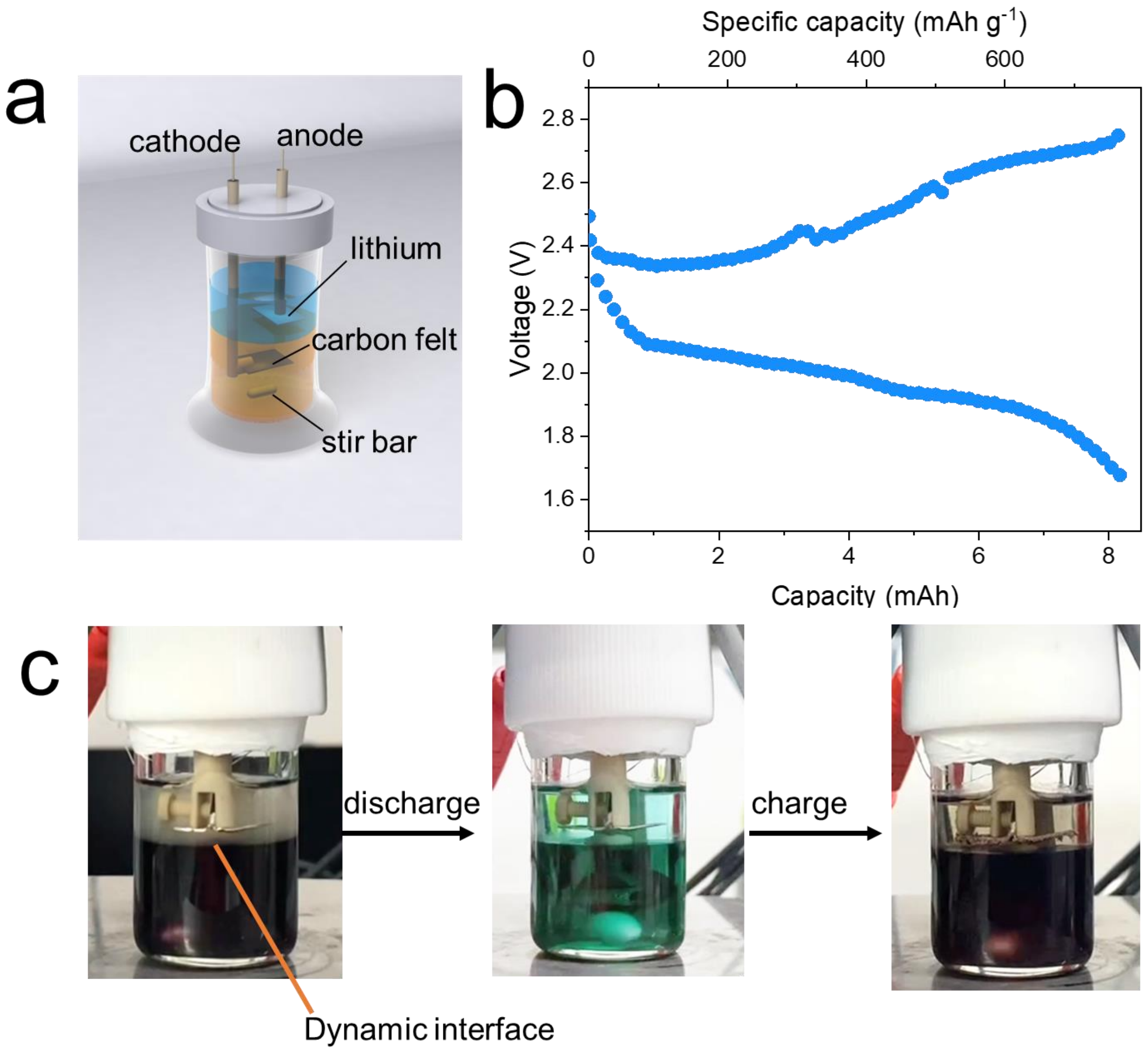


**Supplementary Figure 18.** (a) Schematic illustration of the Li-S BSB with the stirring system. (b) Charge/discharge profiles of Li-S BSBs under stirred environment. (c) Phase interface stability of Li-S BSB during charge and discharge under stirred environment.

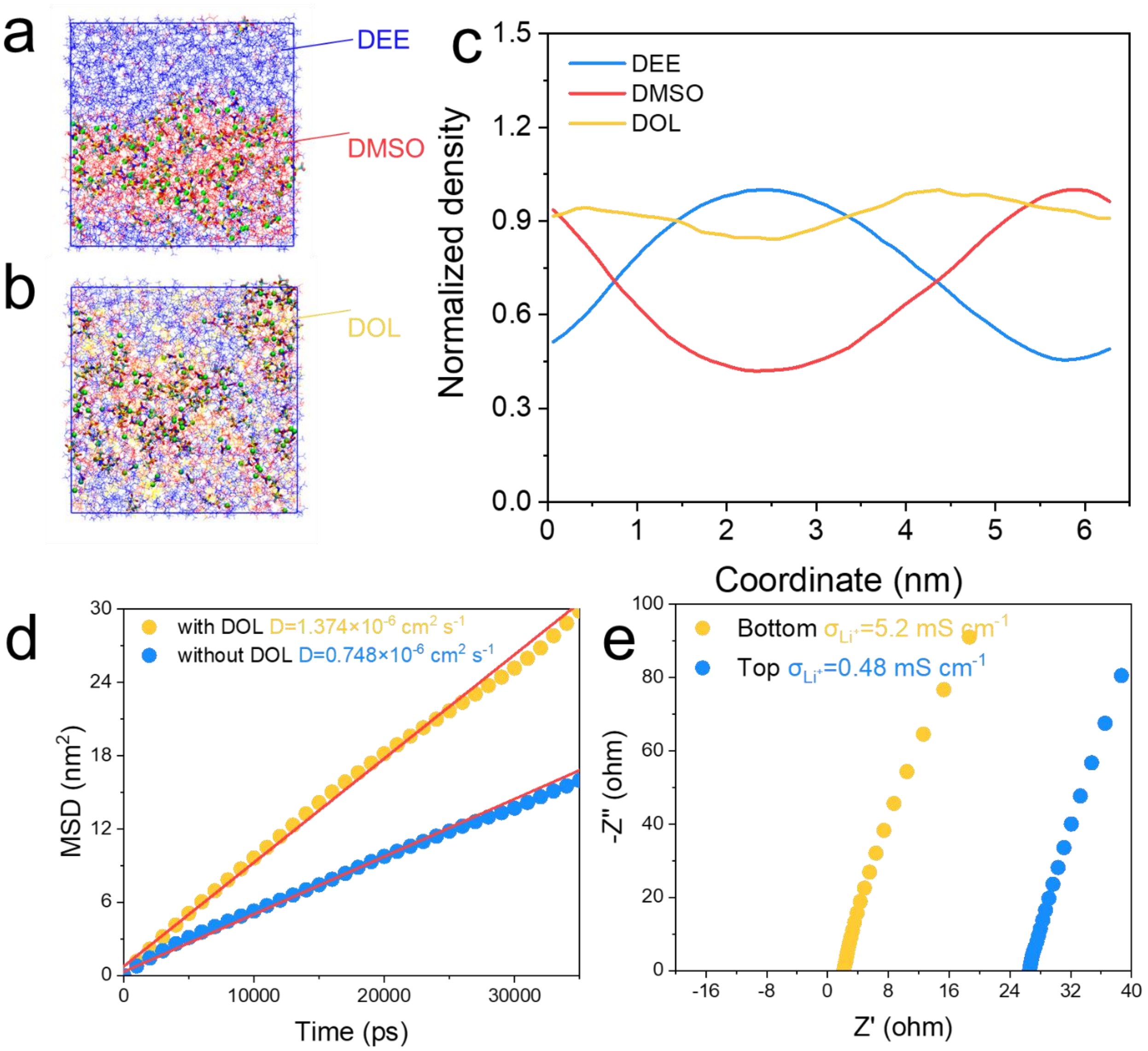


**Supplementary Figure 19. Design consideration of DMSO-DOL-DEE biphasic electrolyte system.** (a)-(b). Snapshots of the MD simulation results of DMSO-DEE system without/with DOL. (c) The normalized spatial density distribution of DMSO, DEE, and DOL in the simulation box. (d) Calculated MSD of $Li^+$ in DMSO-DEE system with/without DOL as a simulation time function. The diffusion coefficient of $Li^+$ was deduced by fitting. (e) $Li^+$ conductivity of the top and bottom phases in DMSO-DEE-DOL biphasic electrolyte system.

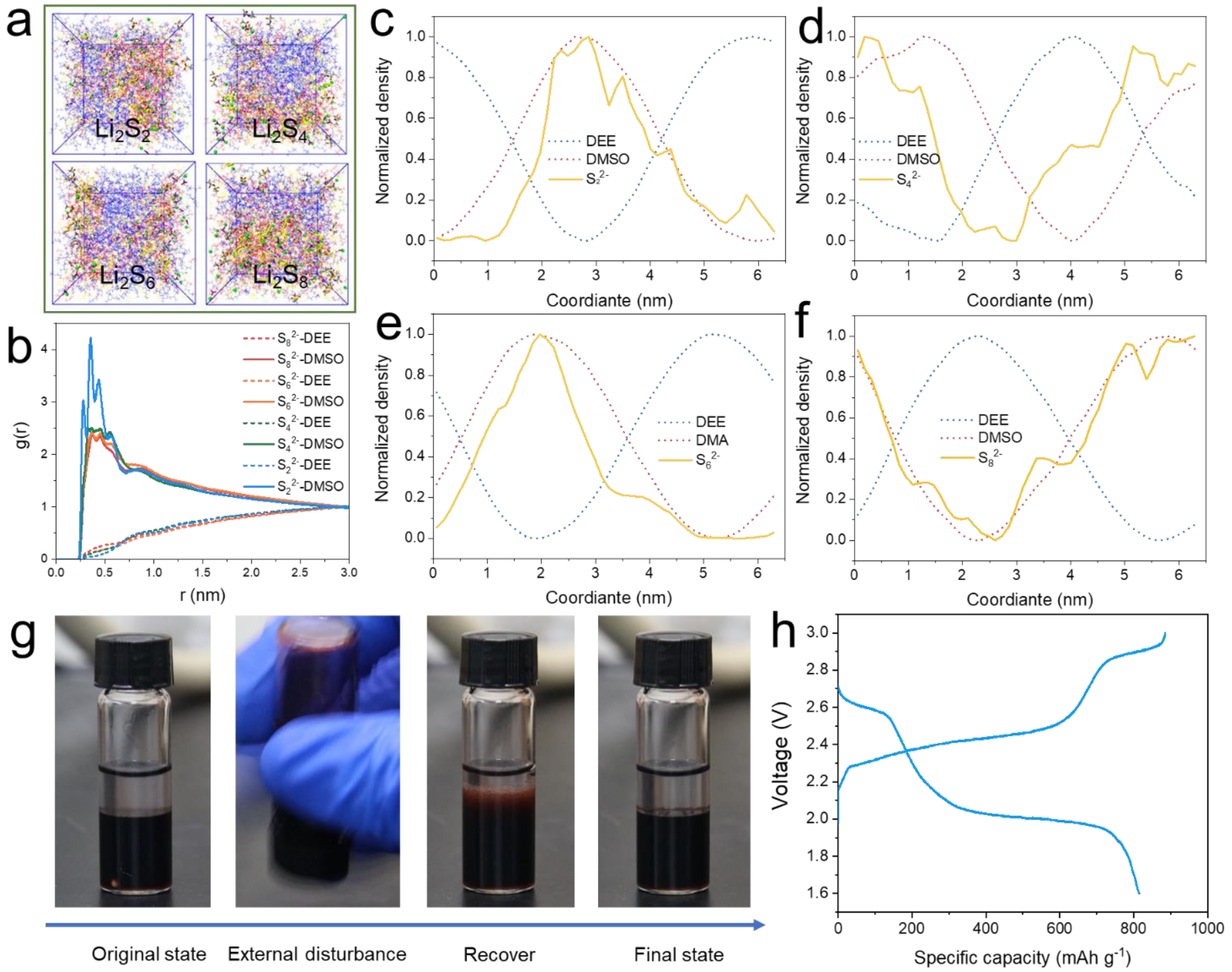


**Supplementary Figure 20. Polysulfide-confinement of DMSO/DEE/DOL biphasic electrolyte system**

(a) Snapshots of the MD simulation results of DMSO-DEE-DOL system containing different lithium polysulfides. (b) RDFs of polysulfide ions between DMSO and DEE. (c)-(f) Normalized spatial density distribution of DMSO and DEE in DMSO-DEE-DOL biphasic system containing various polysulfides. (g) Spontaneous recovery process of DMSO-DEE-DOL biphasic electrolyte system after external disturbance. 10 mM of $Li_2S_8$ was dissolved in the electrolyte. (h) Charge/discharge profiles of a Li-S BSB used DMSO-DEE-DOL biphasic electrolyte system.

Supplementary note:

To demonstrate the universality of our design considerations for nonaqueous biphasic electrolyte systems, we further developed another biphasic electrolyte system formed by the ternary of dimethyl sulfoxide (DMSO), 1,3-dioxolane (DOL), and DEE. DMSO has high permittivity ($\varepsilon_{DMSO}$=48.9) and

high LPSs solubility, enabling it to spontaneously separate from DEE under the salting-out effect of lithium salt and constrain LPSs effectively (Supplementary Figure 19). The addition of DOL can significantly enhance the $Li^+$ conductivity between the bottom and top phases. As demonstrated in Supplementary Figure 19c, the diffusivity of DMSO-DEE system with DOL was estimated to be $1.374\times10^{-6}$ $cm^2$ $s^{-1}$, almost twice as much as without DOL. Finally, the $Li^+$ conductivity of the bottom/top phase in DMSO-DEE-DOL biphasic electrolyte system reaches 5.2 and 0.48 mS $cm^{-1}$, respectively.

As demonstrated in Supplementary Figure 20, this DMSO-DEE-DOL biphasic electrolyte system can also effectively confine different LPS species in the bottom phase and resist external interference. The Li-S BSB based on this biphasic electrolyte system was also fabricated, which delivers a specific discharge capacity of 815 mAh $g^{-1}$, demonstrating its potential in nonaqueous BSBs.

**Supplementary Table 1.** The amounts of different components add to different systems.

| | DEE | DMA | LiTFSI | $LiNO_3$ |
|---|---|---|---|---|
| No. 1 | 1 mL | 1 mL | 287.5 mg | 68.9 mg |
| No. 2 | 2 mL | 1 mL | 287.5 mg | 68.9 mg |
| No. 3 | 3 mL | 1 mL | 287.5 mg | 68.9 mg |
| No. 4 | 1 mL | 2 mL | 287.5 mg | 68.9 mg |
| No. 5 | 1 mL | 3 mL | 287.5 mg | 68.9 mg |
| No. 6 | 2 mL | 1 mL | 287.5 mg | 0 mg |
| No. 7 | 2 mL | 1 mL | 0 mg | 68.9 mg |
| No. 8 | 2 mL | 1 mL | 0 mg | 0 mg |

**Supplementary Table 2.** The integrated area of the signal peak in Supplementary Figure 5c.

| **Top phase** | **Peak position(ppm)** | **Peak area** |
|---|---|---|
| DEE | 3.5 | 9.4 |
| | 1.1 | 14.8 |
| DMA | 2.0 | 1.0 |
| | 2.9 | 2.0 |
| **Bottom phase** | **Peak position(ppm)** | **Peak area** |
| DEE | 3.5 | 1.0 |
| | 1.1 | 1.5 |
| DMA | 2.0 | 1.0 |
| | 2.9 | 2.0 |

**Supplementary Table 3.** The molecule number of each component in different systems for MD simulations.

| | DEE | DMA | $Li^+$ | $TFSI^-$ | $NO_3^-$ | polysulfides |
|---|---|---|---|---|---|---|
| Figure S1a | 962 | 1070 | 200 | 100 | 100 | 0 |
| Figure 1b | 1924 | 1070 | 200 | 100 | 100 | 0 |
| Figure S1b | 1070 | 2886 | 200 | 100 | 100 | 0 |
| Figure S1c | 962 | 2140 | 200 | 100 | 100 | 0 |
| Figure S1d | 962 | 3210 | 200 | 100 | 100 | 0 |
| Figure S1e | 1924 | 1070 | 200 | 200 | 0 | 0 |
| Figure S1f | 1924 | 1070 | 200 | 0 | 200 | 0 |
| Figure S1g | 1924 | 1070 | 0 | 0 | 0 | 0 |
| Figure 2a | 954 | 128 | 11 | 11 | 0 | 0 |
| Figure 2c | 970 | 942 | 189 | 89 | 100 | 0 |
| Figure 3a | 1924 | 1070 | 240 | 100 | 100 | 20 $S_8^{2-}$ |
| | 1924 | 1070 | 240 | 100 | 100 | 20 $S_6^{2-}$ |
| | 1924 | 1070 | 240 | 100 | 100 | 20 $S_4^{2-}$ |
| | 1924 | 1070 | 240 | 100 | 100 | 20 $S_2^{2-}$ |

| | DMSO | DEE | DOL | $Li^+$ | $TFSI^-$ | $NO_3^-$ | polysulfides |
|---|---|---|---|---|---|---|---|
| Figure S17a | 705 | 720 | 0 | 100 | 50 | 50 | 0 |
| Figure S17b | 705 | 720 | 353 | 100 | 50 | 50 | 0 |
| Figure S18a | 705 | 720 | 353 | 120 | 50 | 50 | 10 $S_8^{2-}$ |
| | 705 | 720 | 353 | 120 | 50 | 50 | 10 $S_6^{2-}$ |
| | 705 | 720 | 353 | 120 | 50 | 50 | 10 $S_4^{2-}$ |
| | 705 | 720 | 353 | 120 | 50 | 50 | 10 $S_2^{2-}$ |

**Supporting Data.** Coordinates of structures used in this work

**Top phase in No.2 system**

| | | | |
|---|---|---|---|
| C | 0.10171600 | 0.00718400 | 2.93844800 |
| H | 0.81928500 | 0.78909600 | 2.66399100 |
| H | 0.48602500 | -0.90470900 | 2.46287500 |
| C | -1.23564700 | 0.28429700 | 2.26871200 |
| N | -1.87925600 | 1.43755900 | 2.55850000 |
| O | -1.73912100 | -0.55553900 | 1.49598300 |
| Li | -1.40425200 | -1.11124500 | -0.33111900 |
| C | -3.11223300 | 1.74281900 | 1.84353600 |
| H | -3.62299800 | 0.81063400 | 1.60899000 |
| H | -3.74745000 | 2.36650100 | 2.47962900 |
| H | -2.88924900 | 2.27420700 | 0.91207400 |
| C | -1.21774900 | 2.57247200 | 3.19566100 |
| H | -0.49363800 | 2.22573500 | 3.93243900 |
| H | -0.71827600 | 3.19253500 | 2.44248400 |
| H | -1.97085700 | 3.17207800 | 3.71519000 |
| C | -4.64283400 | 0.93933000 | -1.44319800 |
| H | -4.96012500 | 1.59366100 | -0.62262700 |
| H | -5.54310200 | 0.74978100 | -2.04472400 |
| C | -4.15432400 | -0.38763000 | -0.87096800 |
| N | -4.93263400 | -1.03450800 | 0.03640600 |
| O | -3.07354100 | -0.86660900 | -1.24313000 |
| C | -6.16282900 | -0.48958400 | 0.58974900 |
| H | -6.87886900 | -1.30358700 | 0.74003400 |
| H | -5.98882800 | -0.00376400 | 1.56019800 |
| H | -6.61282900 | 0.23352300 | -0.08872500 |
| C | -4.43344900 | -2.26193100 | 0.65445600 |
| H | -5.27322900 | -2.94255200 | 0.82381000 |

| | | | |
|---|---|---|---|
| H | -3.70979600 | -2.72970200 | -0.01170600 |
| H | -3.93683500 | -2.04493800 | 1.60660600 |
| C | 1.06361300 | -3.90080500 | 0.67324300 |
| H | 2.02192700 | -3.50430800 | 1.01533000 |
| H | 0.32830700 | -3.67663800 | 1.44962100 |
| C | 0.57973500 | -3.15749400 | -0.56108300 |
| O | -0.61628200 | -2.79722400 | -0.61582600 |
| N | 1.42157600 | -2.96210700 | -1.59610000 |
| C | 0.96323000 | -2.26870000 | -2.79671500 |
| H | -0.12259100 | -2.20839000 | -2.78325700 |
| H | 1.38177900 | -1.25872500 | -2.82941100 |
| H | 1.29520000 | -2.82799600 | -3.67906400 |
| C | 2.84908200 | -3.25337900 | -1.58284400 |
| H | 3.09247400 | -3.95028000 | -2.39476100 |
| H | 3.40651200 | -2.32311400 | -1.72594600 |
| H | 3.15354600 | -3.69547200 | -0.63821500 |
| S | 0.29843800 | 1.49497100 | -0.70291700 |
| N | 1.61158000 | 1.45153400 | 0.20220600 |
| S | 2.66000600 | 0.20671100 | 0.08229600 |
| C | 3.95856200 | 0.94962400 | 1.19245200 |
| O | 3.28125000 | 0.04804200 | -1.24064600 |
| O | 2.22023200 | -1.01144700 | 0.78302300 |
| O | -0.17313100 | 0.16502200 | -1.15495000 |
| F | 3.45939200 | 1.16995400 | 2.41475700 |
| F | 4.42304500 | 2.09211100 | 0.69216300 |
| F | 4.96267500 | 0.07082100 | 1.28840500 |
| O | -0.67118400 | 2.41291500 | -0.09433000 |
| C | 0.76615900 | 2.32349500 | -2.31094500 |

| | | | |
|---|---|---|---|
| F | -0.35087000 | 2.54574500 | -3.01593000 |
| F | 1.36807000 | 3.48822300 | -2.06642800 |
| F | 1.58101300 | 1.54407700 | -3.02219800 |
| H | -3.89226930 | 1.42683960 | -2.02961681 |
| H | 0.04046864 | -0.09987793 | 4.00131511 |
| H | 1.11331779 | -4.95767774 | 0.51371420 |

## Bottom phase in No.2 system

| | | | |
|---|---|---|---|
| C | -5.83473135 | 2.93534415 | -4.15053473 |
| H | -6.32940063 | 2.77416769 | -5.08681032 |
| H | -4.82693668 | 3.25523019 | -4.33124701 |
| H | -6.35800267 | 3.69136474 | -3.59687009 |
| C | -5.82350304 | 1.62258681 | -3.33952889 |
| O | -5.83363787 | 1.66346765 | -2.08196730 |
| N | -5.80384377 | 0.32224987 | -4.02955710 |
| C | -6.47119094 | -0.68275387 | -3.19026443 |
| H | -6.45725653 | -1.63127782 | -3.68793503 |
| H | -5.95711403 | -0.76128072 | -2.25611528 |
| H | -7.48579254 | -0.38873814 | -3.01453833 |
| C | -4.41574241 | -0.08775477 | -4.27194992 |
| H | -4.40378833 | -1.03379913 | -4.77275900 |
| H | -3.92972366 | 0.64154974 | -4.88209870 |
| H | -3.90380772 | -0.17002754 | -3.33720559 |
| C | -3.78262001 | 4.77355587 | -2.93390117 |
| H | -3.38719268 | 5.68694351 | -3.32872199 |
| H | -4.85219177 | 4.80840284 | -2.96445566 |
| H | -3.43483138 | 3.94920899 | -3.52479617 |
| C | -3.31411469 | 4.59384501 | -1.47687227 |
| O | -4.03835765 | 3.96885003 | -0.65729541 |
| N | -2.02328408 | 5.14860376 | -1.04392961 |
| C | -0.96985887 | 4.13373918 | -1.21061444 |
| H | -0.02791033 | 4.53845858 | -0.90332718 |
| H | -1.20059017 | 3.28248655 | -0.61184027 |
| H | -0.91302631 | 3.84194961 | -2.23651278 |
| C | -2.10729475 | 5.53817773 | 0.37447273 |
| H | -1.16725466 | 5.94063612 | 0.69326375 |
| H | -2.87010357 | 6.28021004 | 0.49525746 |
| H | -2.34600690 | 4.67639954 | 0.96767625 |
| Li | -5.74305986 | 3.33479138 | -1.18701665 |
| N | -7.13735969 | 5.06006601 | 0.29216063 |
| O | -7.30429521 | 4.32376264 | -0.84145079 |
| O | -6.88987199 | 4.23284472 | 1.34416580 |
| O | -6.08930076 | 5.91204427 | 0.12842514 |
