# Supplementary material for: Exploiting nonaqueous self-stratified electrolyte systems toward large-scale energy storage
Source: Nat Commun. 2023 Apr 20;14:2267. doi: 10.1038/s41467-023-37995-8 (PMC10119102; doi:10.1038/s41467-023-37995-8)
Supplement: Supplementary file 3 — Description of Additional Supplementary Files [file 41467_2023_37995_MOESM3_ESM.pdf]

Description of Additional Supplementary Files

File Name: Supplementary Movie 1
Description: Test of resistance to external force disturbance of DMA-DEE biphasic system.

File Name: Supplementary Movie 2
Description: Phase interface stability of Li-S BSB during charge and discharge under stirred environment.

File Name: Supplementary Movie 3
Description: Test of resistance to external force disturbance of DMSO-DEE-DOL biphasic system.
